# Long-term outcomes of tonsillectomy in children with periodic fever, aphthous stomatitis, pharyngitis, adenitis (PFAPA) syndrome

**DOI:** 10.1186/1546-0096-13-S1-O78

**Published:** 2015-09-28

**Authors:** L Broderick, D Carvalho, A Magit, W Jiang, S Leuin, M Bothwell, D Kearns, S Pransky, H Hoffman

**Affiliations:** 1 University of California, San Diego, La Jolla, San Diego, CA, USA; 2 Rady Children's Hospital-San Diego, San Diego, CA, USA

## Introduction

Periodic fever, aphthous stomatitis, pharyngitis and adenitis (PFAPA) syndrome is an inflammatory disorder of childhood classically characterized by recurrent fevers, pharyngitis, stomatitis, cervical adenitis and leukocytosis. Little is known about the true incidence, natural course, pathogenesis, and appropriate therapy in this recently described syndrome. While the mechanism is unclear, previous studies have shown that tonsillectomy can be a therapeutic option with improvement in quality of life in many patients with PFAPA, but long-term clinical follow up is lacking.

## Objective

To evaluate the long-term response of tonsillectomy in patients with PFAPA syndrome treated at a tertiary care center in San Diego, CA.

## Methods

We initiated a prospective cohort study in 2008 to better understand the natural history of PFAPA in children treated at a tertiary care center in San Diego, CA. Any patient aged 1-17 years, seen in the Rady Children's Hospital-San Diego Allergy/Immunology clinics, diagnosed clinically with PFAPA syndrome and undergoing tonsillectomy (with or without adenoidectomy) were eligible for inclusion. Patient data was collected on over 200 children with recurrent fevers including 94 patients with PFAPA under an IRB-approved protocol. Using patient charts and a standardized questionnaire, demographic data, including age, gender, and ethnicity, clinical profiles (presence of symptoms, fever profile, treatments) and detailed family histories were obtained from patients seen in a children's hospital-based clinic over a 7-year period. A subset of samples (10 PFAPA and 10 control) underwent 16S rDNA profiling.

## Results

To date, 63 patients with PFAPA and 11 patients with other non-infectious recurrent fevers have undergone tonsillectomy. Forty-four patients with PFAPA syndrome have had complete resolution of symptoms after surgery, with the time to resolution of symptoms post-tonsillectomy approximately 2 months (range 1-11 months). The average length of follow up is 31.6 months (range 1-58 months). In 3 patients, there has been a relapse of symptoms, defined as fevers persisting for more than 6 months, which remain responsive to medical therapy. Post-operatively, tonsils from patients with PFAPA are notably smaller and grossly friable. No granulomas or abscesses were noted on histological examination. While small differences exist in the tonsillar microbiome of PFAPA patients compared to controls, these taxonomic groups were only very low abundance, and were not outside the range of normal flora observed in the Human Microbiome Project.

## Conclusions

Our cohort of patients demonstrates clinical characteristics consistent with PFAPA. This study demonstrates that tonsillectomy is an effective surgical treatment option for management of children with PFAPA syndrome.

